# Gel Shrinkage in Discontinuous Electrophoresis: How to Stabilize the Electrolyte Boundary in Epitachophoresis – Part 2 – Nongel Solid Support

**DOI:** 10.1021/acsomega.5c09625

**Published:** 2026-01-22

**Authors:** Vanda Kocianová, Ivona Voráčová, Doo Soo Chung, František Foret

**Affiliations:** † Institute of Analytical Chemistry of the CAS, Veveří 97, 602 00 Brno, Czech Republic; ‡ Department of Biochemistry, Faculty of Science, Masaryk University, Kamenice 5, 625 00 Brno, Czech Republic; § Department of Chemistry, Seoul National University, Seoul 08826, Republic of Korea

## Abstract

In the first part of this study, we have examined the shrinkage of hydrophilic gels during epitachophoresis, an isotachophoresis-like discontinuous electrophoretic technique, applied to concentrate DNA samples. In the present work, we evaluated selected solid porous media (sponges, nanofibers, foamed polymers, membranes, and structured inserts) as alternative anticonvective media. All materials were assessed based on zone shape, ease of creating the boundary between the leading and trailing electrolytes, and the DNA recovery. While nanofibers and most sponges resulted in poor separation or high analyte adsorption, mechanically supported agarose gels and filtration membranes provided sharp dye zones and high DNA recovery. Foamed polymers, especially plasma-treated ultrahigh molecular weight polyethylene, showed the best overall performance. Some rigid open structures (e.g., silica columns or nylon nets) demonstrated potential for large analytes but require further optimization. These results highlight key design considerations for robust, scalable epitachophoresis devices for preparative DNA concentration using solid-state stabilization media.

## Graphical abstract

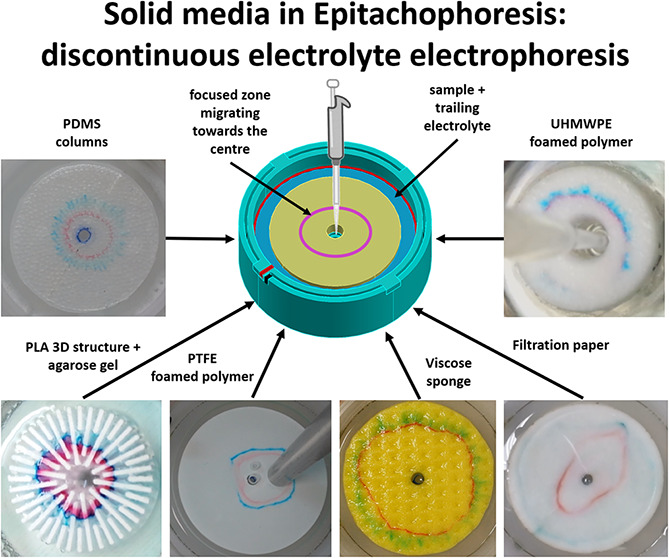

## Introduction

Sample concentration and purification are key requirements for successful DNA analysis. Epitachophoresis (ETP) is an emerging electrophoretic technique for the separation, preconcentration, and purification of biomolecules. It is based on the principles from isotachophoresis—utilizing a discontinuous system with a leading electrolyte (LE) of high electrophoretic mobility and a terminating electrolyte (TE) of low electrophoretic mobility—to focus analytes whose electrophoretic mobilities fall between those of the LE and TE.^1^ While conventional isotachophoretic separations are typically carried out in capillaries, ETP employs planar, radial platforms that enable concentrated analyte collection at the center of circular devices.^2^ For processing multimilliliter samples, a stabilizing medium that prevents convection mixing during separation is critical. In previous work, hydrogels were investigated as stabilizing media owing to their biocompatibility, tunable porosity, and established use in electrophoretic and bioanalytical applications.^2–5^ However, the most common agarose gels revealed technical limitations, particularly poor structural integrity and susceptibility to shrinkage during runs, which compromised the electrophoretic performance. These limitations motivated the exploration of alternative materials capable of providing mechanical stability without interfering with separation. Solid porous media, widely used in chromatography, represent a possible alternative; however, while chromatographic media are engineered for specific interactions with analytes, ETP demands an inert, noninteractive phase that enables separation based solely on electrophoresis. Solid materials, previously applied in size-exclusion chromatography, such as silica gel, Sephadex, and alumina, were repurposed for investigation in this context.^6,7^ Monolithic and sintered porous polymers have received growing attention as alternatives to particle-packed media due to their continuous porous networks and mechanical robustness.^8^ Sintered polymers, in particular, are manufactured by heat- or pressure-induced fusion of powdered polymer particles without reaching their melting point, producing a matrix of interconnected pores.^9^ This architecture enables consistent flow paths and has been applied in filtration and microextraction. Despite their structural advantages, many solid polymers are inherently hydrophobic, which limits their compatibility with aqueous buffer systems and impairs electrolyte uptake.^10^ Various surface modification strategies were evaluated to increase hydrophilicity, including low-pressure plasma treatment,^11^ oxidative modification using piranha solution,^12^ and silanization.^13^ Additional media such as paper membranes, nanofiber mats,^14^ and sponge materials were also considered due to their inherent hydrophilic properties and ease of integration into small-scale devices.

This study evaluates various solid stabilization media for epitachophoretic separation. We assess their physical structure, surface properties, compatibility with the LE/TE system, and overall performance in dye and DNA separations. The goal is to identify robust, inert, and hydrophilic stabilizing materials suitable for potential application in single-use epitachophoretic cartridges.

## Experimental/Materials and Methods

### List of Chemicals and Materials

Chemicals used for electrolyte preparation were l-histidine hydrochloride monohydrate (98.5–101.0%), l-histidine (98.5–101.0%) both from PanReac AppliChem, ITW Reagents (Karlsruhe, Germany). *N*-tris­(hydroxymethyl)­methyl-3-aminopropanesulfonic acid (TAPS, 99.5%) and tris­(hydroxymethyl) aminomethane (TRIS, 99.9%) were from Carl Roth (Karlsruhe, Germany), (1,3-bis­[tris­(hydroxymethyl) methylamino]­propane) (Bis-Tris propane; BTP, 99%) from Sigma-Aldrich (St. Louis, MO), and hydrochloric acid (35%) from Lach-Ner (Brno, Czech Republic).

Patent blue V sodium salt, and 1,8-dihydroxy-2-(4-sulfophenylazo)-naphthalene-3,6-disulfonic acid trisodium salt (SPADNS, ≥80%), both from Sigma-Aldrich, were used for visual observation of the moving LE/TE boundary. SYBR Gold for DNA fluorescent labeling was from Invitrogen (Carlsbad, CA). The 1 kb DNA ladder was obtained from New England BioLabs (Ipswitch, MA). All solutions were prepared in deionized water (NEPTUNE Purite Ultimate, ONDEO, UK).

The foamed polymers provided by POREX (Fairburn, GA), Roche (Indianapolis, IN), and SCI Scientific Commodities (Tucson, AZ) are listed in Table 1. Nanofiber sheets were kindly provided by Prof. Dalibor Šatínský, Pharmaceutical Faculty, Charles University in Hradec Králové, Czech Republic. Membrane-based stabilization media were poly­(ether sulfone) (PES) membrane filter (0.22 μm, hydrophilic, Fisherbrand, Pardubice, Czech Republic), nylon empty tea bag (Wish Tea Bag, China), and Durapore microfiltration membrane (polyvinylidene fluoride (PVDF), 0.45 μm, Millipore, Billerica, MA). Silicone columns were prepared using a laboratory-made mold and Sylgard 184 (Dow Coming, Corning, NY). Gel-blotting paper (GB 005, Whatman, Dassel, Germany) and foamed polymers were cut to the proper shape using a laser cutter (Nova 24, Thunder Laser, Narran Laser Precision, Brno, CZ). Polyurethane (PU) sponge and viscose sponge cloth (Niteola) were from Melittrade, Praha, Czech Republic. For the hydrophilic treatment of polymers, a low-pressure O_2_ plasma system (Zepto, semiautomatic, 40 kHz, 0–100 W, Diener Electronic, Ebhausen, Germany) or a piranha solution was used. The piranha solution was prepared by mixing sulfuric acid (96%, Lach-Ner) and hydrogen peroxide (30%, Penta Chemicals Unlimited, Hostivař, Czech Republic), in a 3:1 volume ratio. Additional surface modifications included treatment with 3-(aminopropyl)­triethoxysilane (APTES, Sigma-Aldrich, Schnelldorf, Germany) and 1% solution of hydroxymethylethyl cellulose (Villa Labeco, Spišská Nová Ves, Slovakia).

**Table 1:** Summary of Solid Stabilization Media for ETP^a^

no.	material	structure	producer	modification	*c*_LE_ (mM)	*c*_TE_ (mM)	dyes zone	DNA recovery (%)
1	PPS polarized positive	nanofiber	Prof. Šatínský	O_2_ plasma	10	10	N	
2	PA6			O_2_ plasma			N	
3	Polyimide Ac			O_2_ plasma			N	
4	PE 2%			O_2_ plasma			N	
5	40% PP, 60% PHB			O_2_ plasma			N	
6	PLA star +0.2% NEEO agarose	insert + filling	Our laboratory		20	10	Y	70
7	PLA star +10% corn starch				100	10	Y	10
8	5% starch, railing insert						N	
9	Railing insert + Sephadex 200						N	
10	Railing insert + silicagel 1–3 mm						N	
11	Railing insert + filtration paper + Al_2_O_3_						N	
12	PDMS columns	structure		O_2_ plasma	20	10	N	70
13	Microsponge	sponge	Aliexpress	0.1% MHEC		50	N	
14	PE	sponge	POREX			10	N	
15	PE	foam			100	10	N	
16	PE	sponge					N	
17	PE	sponge					N	
18	PE	sponge		O_2_ plasma	20	10	N	
19	Nylon/PP	nonwoven fabric			100	30	N	
20	Nylon	net	Wish Tea Bag		100	10	Y	85
21	PU	sponge	Niteola		50	20	Y	70
22	PU	sponge	unknown		20	10	Y	14
23	viscose	sponge	Niteola		20	10	Y	15
24	PES	membrane	Thermo Fisher Scientific		100	20	Y	90
25	PVDF 0.45 μm		Millipore		100	10	Y	100
26	Filtration paper	fibers	Whatman		20	10	Y	23
27	PG 20 μm	foamed polymer	Roche	O_2_ plasma	20	10	N	
28	PE 5 μm			O_2_ plasma			N	
29	UHMWPE7–12 μm			O_2_ plasma + MHEC			Y	76
30	PP 80–155 μm			O_2_ plasma			N	
31	PTFE 10–45 μm			O_2_ plasma + MHEC			Y	7
32	PE 30 μm	foamed polymer	SCI Scientific Commodities	hydrophilic	100	10	Y	90
33	UHMWPE 50 μm			O_2_ plasma	100	10	N	
34	UHMWPE 50 μm			piranha	100	20	N	
35	UHMWPE 50 μm			APTS	100	10	N	

a Experimental conditions: experiments 1–5, LE: HCl/BTP, TE: TAPS/BTP; experiments 6–35, LE: HCl/His, TE: TAPS/Tris.

## Device Setup

The inserts were fabricated from polylactic acid (PLA) filament (PM Filaments, Haňovice, Czech Republic) using an Original Prusa i3MK3S 3D printer (Prusa Research, Prague, Czech Republic) with a 0.25 mm nozzle and a step height of 0.1 mm. Fusion 360 (Autodesk, San Rafael, CA) was used to design the desired structures. PLA was selected for its negatively charged surface carboxyl groups to minimize sorption of the negative sample ions.^3^ For testing, the 3D printed inserts were filled with one of the following substances: aluminum oxide (for chromatography, neutral, Brockmann I, 50–200 μm, 90 A, Acros Organics, Geel, Belgium), corn starch (Natura, Nový Bydžov, Czech Republic), Sephadex G-200 (Pharmacia Fine Chemicals, Uppsala, Sweden), silica gel 60 (1.0–3.0 mm, Carl Roth), or agarose NEEO (no electroendosmosis) ultra quality ROTIGarose (Carl Roth).

Two epitachophoretic devices were employed:

- A large device (95 mm diameter), fabricated by injection molding, suitable for sample volumes of several milliliters with correspondingly larger stabilizing media.
- A miniETP (25 mm diameter), designed for smaller-scale studies and barrier-type stabilization.

Both devices consisted of nested reservoirs with electrodes and semipermeable membranes. Stabilizing media were positioned at the LE/TE interface. Power supplies were operated in either constant power mode (2 W for the large device, 0.5 W for miniETP) or constant voltage mode (150 V). Separation times ranged from 5 to 50 min, depending on the device and medium. A stainless steel wire (stainless steel 1.4301, Hobby Dráty, Czech Republic) ring was used as the cathode (negative electrode), and 0.3 mm Pt wire (SAFINA, Vestec, Czech Republic) served as the anode. Alligator clips were used for electric connections. Samples were collected in Slide-A-Lyzer mini dialysis cups (2000 Da MWCO, Thermo Fisher Scientific, Waltham, MA). The device setup is shown in Figure 1.

**Figure 1:**
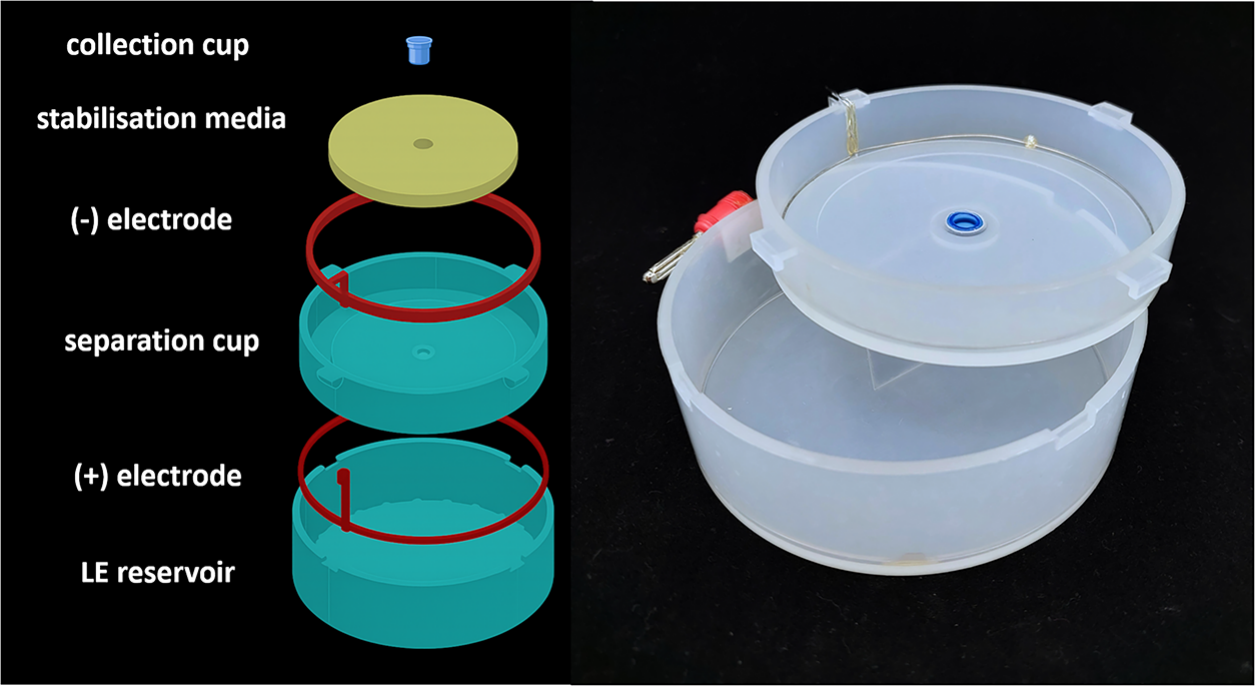
Schematic and photograph of a large epitachophoretic device, 95 mm diameter

The smaller device with a diameter of 25 mm, called miniETP, was designed and manufactured by Protolab of Roche (Figure 2). The device is composed from three main parts: the LE reservoir – bottom part, the top separation cup with electrodes and the inset with stabilization media. The top cup consisted of a main body with an ID of 25 mm, an OD of 30 mm, a height of 15 mm, and a central hole of 8 mm, into which a cylindrical insert holding the stabilization medium was placed. The main body and insert part were made by 3D printing from PLA (Figure 2) or by milling from polycarbonate, depending on the insert used. The O-ring used to seal the main body with the inset was from McMaster (Elmhurst, IL). The cathode, located on the inside wall of the top cup, was made from a stainless steel sheet (2 mm, Smalley, Lake Zurich, IL), and cut to an “L-shape.” The horizontal part of the L-shape electrode was taped inside the main body of the device, and the vertical extension was glued to the side holder, enabling connection with the power supply by an alligator clip. The flexible graphite sheet/foil with adhesive back (thickness 0.005 in., 99% carbon, Amazon.com, Seattle, WA), placed on the outer wall of the top cup, served as the anode, also connected via an alligator clip. The bottom separation membrane for sample collection was constructed from several layers such as sandwich: bottom of the top cup, a double-sided adhesive (DSA) foil (Acrylic Adhesive 300LSE, 3M, Maplewood, MN), cellulose acetate membrane (500 Da, Harvard Apparatus, Holliston, MA), a second DSA layer (3M), and final layer of GelBond film sheet (Lonza Rockland, Rockland, ME). All layers had a circular shape with a diameter of 15 mm and an 8 mm central hole, except for the semipermeable cellulose membrane without the central hole. A 6-well plate (VWR, Radnor, PA) was used as the bottom reservoir.

**Figure 2:**
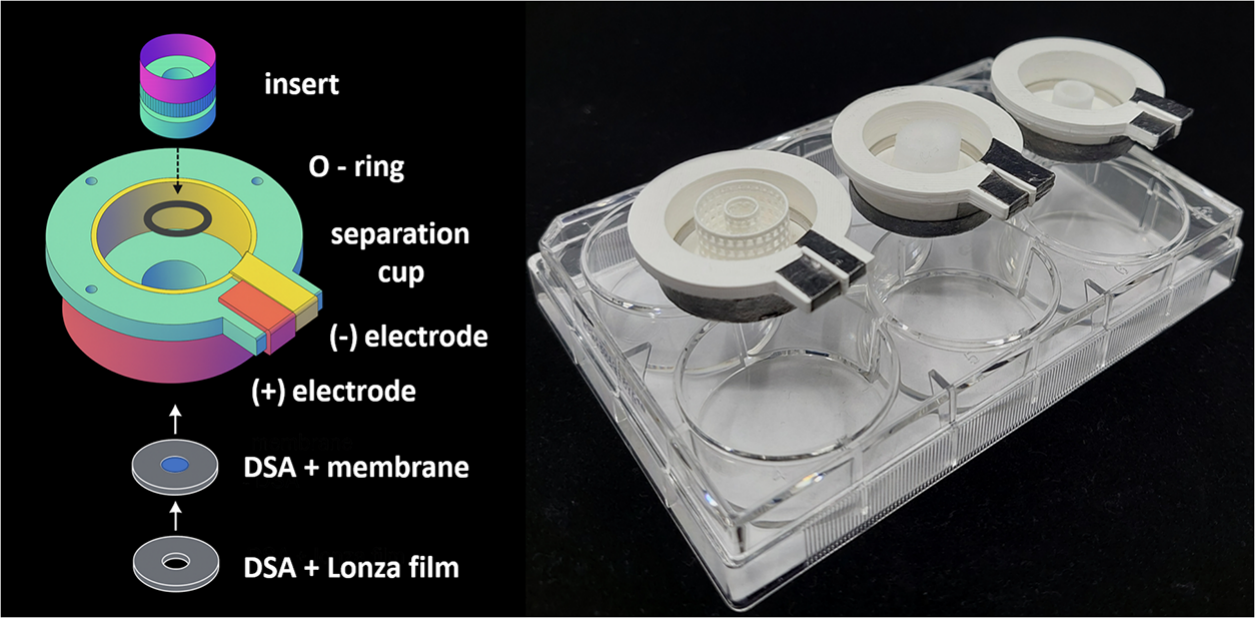
Schematic and photograph of a miniETP epitachophoretic device, 25 mm diameter – left. Three different separation inserts are shown in the separation cups placed on the top of the six well leading electrolyte reservoirs – right.

The complete setup of the miniETP device consisted of a 6-well plate serving as the LE reservoir, a tray keeping the top cups in place, and the top cup with both electrodes attached, where the separation takes place (Figure 2).

The solid media were attached to the ETP device using various methods. For the large ETP device, the separation media were cut to the appropriate size and affixed to the top cup with DSA. In the case of the miniETP, several strategies were applied because of the variability of the media used. Thin sheets (e.g., nylon, PES, and PVDF) were inserted into the central part of the main body of miniETP and fixed with an O-ring or glued with DSA. Solid media (e.g., sponges, 3D-printed inserts) were shaped to fit the central part of the main body without further attachment.

Several solid media we tested were hydrophobic. This complicated or even disabled the media to suck in the LE. Therefore, the surface modification to hydrophilic was necessary. We used several strategies to increase the hydrophilicity of the media surface. In the first approach, we applied O_2_ plasma for 15 min. This proved to be the easiest and most effective option. However, this modification was not permanent and had to be applied immediately before each experiment. Another modification was using piranha solution.^12^ The polymers were first treated with O_2_ plasma for 5 min to induce hydrophilicity, then submerged in piranha solution for 20 min, and washed with deionized water. This treatment resulted in permanent hydrophilic surfaces. A similar protocol was applied for silanization: 5 min plasma treatment followed by 20 min immersion in 2% APTES solution and washing with deionized water. A surfactant, 0.1% MHEC, was also tested. Adding MHEC to the LE was not sufficient enough to enable suction of LE into the solid medium. On the other hand, the addition of MHEC to LE improved the separation in some cases. Two electrolyte systems with different counterions were prepared: LE (20–100 mM HCl titrated with His to pH 6.4, or 10 mM HCl titrated with BTP to pH 7.7) and TE (10–50 mM TAPS titrated with Tris to pH 8.3, or 10 mM TAPS titrated with BTP to pH 7.7).

Before each experiment, the bottom cup was filled with LE. The top cup was assembled with a membrane—either a collection cup or the layered membrane at the bottom, depending on the device used—and a stabilizing medium was attached. The medium was soaked in LE, and the collection area was also filled with LE. The top cup was nested inside the bottom cup. The TE mixed with the sample was poured in, and the power supply was set to a constant power of 2 W or a constant voltage of 150 V for the large ETP device and a constant power of 0.5 W for the miniETP device. Separation times were approximately 25–50 min for the large ETP device, and 5–15 min for miniETP, depending on the stabilizing medium. After separation, the sample was collected from the center of the device with a pipette.

Two organic dyes and 1kb DNA ladder with 15 DNA fragments from 1kb to 15kb were used. The molecular weight and electrophoretic mobility of SPADNS are 570.4 g·mol^–1^ and 55 × 10^–9^ m^2^·V^–1^·s^–1^, respectively. For Patent Blue, the values are 496.4 g·mol^–1^ and 32 × 10^–9^ m^2^·V^–1^·s^–1^, respectively. Both dyes have a molecular weight lower than the molecular weight cutoff of the membranes used. Therefore, they can′t be used for recovery determination. The DNA fragments have very similar electrophoretic mobilities for short and long fragments.^15^ The dyes perfectly fit the range of DNA fragments’ mobility.

The dyes were detected optically, and video recordings and photographs were acquired using a mobile phone (Samsung Galaxy S9 Plus). DNA fragments were labeled with SYBR Gold, and the resulting fluorescent complexes were detected with a blue UV LED. The fluorescence was captured through a yellow-orange photographic filter (040 Yellow-Orange B&W, Schneider Optics, Hauppauge, NY) using the mobile phone. The DNA recovery was calculated as the amount of DNA loaded (1 μg) divided by the amount of DNA collected. The amount of collected DNA was calculated from the collected volume and the DNA concentration in the collected solution. The DNA concentration was determined by a Qubit fluorimeter (Invitrogen) according to the manufacturer’s instructions, using Qubit dsDNA Quantification Assay Kits (Invitrogen).

## Results and Discussion

ETP operates in a discontinuous electrolyte system; therefore, the stabilization of the boundary between the two electrolytes is necessary. Several approaches can be used to stabilize the LE/TE boundary, such as gels, 3D-printed structures, membranes, and solid materials with large pores. The use of a solid medium simplifies both the preparation and manipulation of the device before the experiment. From a practical standpoint, maintaining a stock of solid stabilizing media is more convenient than preparing gels before each run. The replacement of the gel with a solid support is important mainly in the case of mini ETP, where narrow and tall gels have to be prepared. Such a gel is hard to prepare and transfer from mold to the device, even when highly concentrated gels are used. They also tend to have low mechanical stability, deformation, shrinkage and collapsing. There are several important requirements for a solid medium suitable for LE/TE stabilization in ETP: chemical inertness, mechanical stability, a hydrophilic surface, and minimized electroosmosis during the ETP run.

Table 1 summarizes the tested materials and the results of the concentration and separation of dyes and DNA. In the separation of the two dyes, zone formation, sharpness, and dye adsorption to the medium were evaluated. The formation of dye zones for each stabilizing medium studied is shown in Figure 2. The experiment numbers in Table 1 correspond to the photograph numbers in Figure 2. When the dye separation was successful, experiments with the DNA ladder were performed to observe its behavior in the medium. DNA recovery was tested after selecting the suitable medium based on the dye separation.

The nanofibers (experiments 1–5) proved unsuitable; the dye zones were smeared, and the separation was unsuccessful. The media with supportive constructions and fillings (experiments 6–11) exhibited variable results, strongly dependent on the chosen filling, less on the construction shape. The NEEO agarose and corn starch gels showed promising results for dye separation; however, in the case of corn starch, the DNA recovery was low. An advantage of using a 3D-printed star insert with agarose gel (experiment 6), instead of agarose gel alone, is that it allows the use of very low gel concentrations. The 3D-printed construction supports very low gel concentrations solutions and provides high DNA yields without the shrinkage observed in more concentrated gels, as reported in Part I.^3^ Sephadex and aluminum oxide fillings were difficult to load with the LE. In addition, their anticonvective properties were weak, leading to liquid mixing. Silica gel fillings absorbed the dyes, and the separation could not be completed.

An array of PDMS columns (experiment 12) represented a highly open structure that is indispensable for extremely large analytes such as viruses or bacteria. Such analytes are difficult to separate using other stabilization media with pore sizes similar to or smaller than the analyte size. The insert consisted of an array of columns 3 mm high, 1 mm in diameter, and spaced 1 mm apart. The size of the inset was 90 mm with a 5 mm central hole for analyte collection. The LE/TE border was stabilized only by capillary forces; consequently, the boundary was imperfectly stabilized, and the analyte zones were broadened (Figure 3, experiment 12). Nevertheless, similar to our previous devices with trapezoidal channels,^16^ the DNA recovery reached 70%. The presence of a semipermeable membrane in the collection cup with an appropriate MWCO contributed to the high recovery. To increase the hydrophilicity of the PDMS column array to allow proper filling with the LE, O_2_ plasma surface modification was necessary. A nylon net (experiment 20) presents another interesting option for very large analytes due to its open structure and high DNA recovery (85%). However, it requires further exploration, as under our conditions, the net was too complex to anchor or glue into the device.

**Figure 3:**
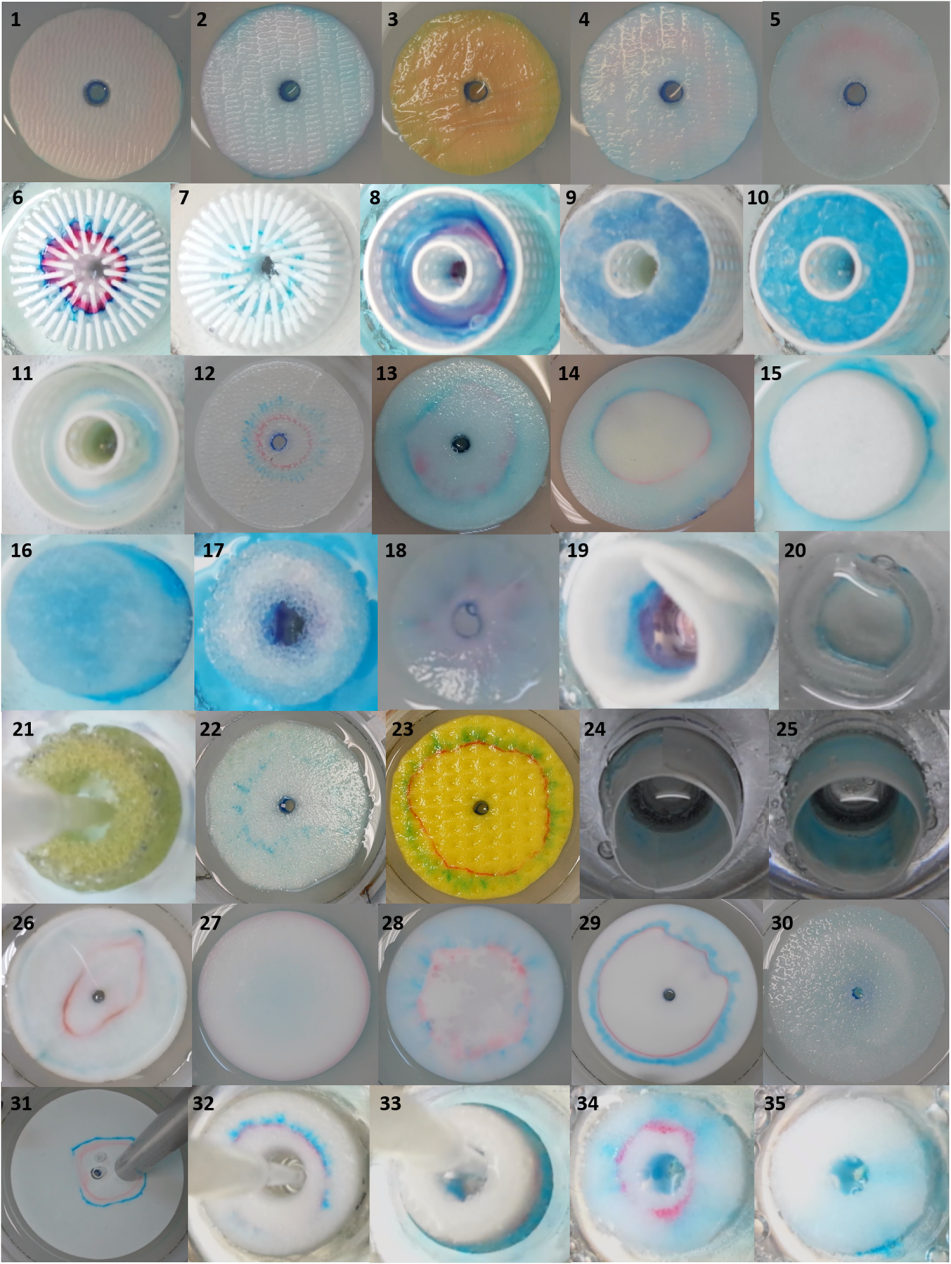
Epitachophoretic separations of dyes with different stabilizing media. The photograph numbers correspond to the stabilization medium numbers listed in Table 1.

Sponges (experiments 13, 14, 16, 18, 21, 23) appeared to be the easiest option for introducing LE into the stabilizing media, as they naturally absorb liquids. Unfortunately, the tested materials proved unsuitable, since a large portion of the dye sample remained within the sponges and did not migrate. This could be due to the material structure, where pores formed cavities with nonuniform lengths and directions, or nonconnected pores, or too strong interactions were present. The only successful case was the PU sponge barrier in miniETP (experiment 21), where the pores are big, and the separation path was relatively short, ensuring connectivity between them. In this configuration, the dyes and DNA were separated efficiently, with DNA recovery at 70%. This was confirmed when a PU sponge was used in a large device, yielding unsatisfactory DNA recovery. Better dye separation was obtained with the viscose sponge (experiment 23), but the DNA recovery was low (15%).

When filtration membranes (experiments 24 and 25) were used in the miniETP device as stabilization barriers, they were highly effective, providing satisfactory dye separation and high DNA recovery (up to 100%). However, they introduced a new problem of lowering the LE level inside the collection cup during the separation process. This effect was related to the surface charge of the membranes, which caused the electroosmotic pumping of the liquid. Consequently, the LE in the collection cup had to be replenished during the separation.

We also tested filtration paper (experiment 26), a well-known separation medium from the early days of chromatography and paper electrophoresis. As shown in Figure 3, experiment 26, the zone of the red dye was sharp, while the blue dye was adsorbed, and the DNA recovery was low. Nevertheless, filtration paper may still serve as an inexpensive material for many analytes tested in the past.^17^ One disadvantage is its low volume, which limits the sample load. When used as a barrier in the miniETP, this limitation no longer poses a concern.

Foamed polymers (experiments 15 and 27–35) were the most successful branch of solid media research for large ETP. The results depended mainly on surface treatment, and to a lesser extent, on shape and on the material used. The main focus was on polyethylene (PE) based materials, PE and ultrahigh molecular weight polyethylene (UHMWPE). Unless the polymer was manufactured as hydrophilic (experiment 32), surface treatment was required. The easiest and most successful method was O_2_ plasma treatment. Treatment with piranha solution (experiment 34) made the surface hydrophilic and enabled dye separation. However, separation of the labeled DNA ladder with SYBR gold was obstructed, and the majority of the DNA stayed adsorbed onto the inset. Silane modification (experiment 35) substantially altered the surface chemistry, and dye separation was not possible. PG (20 μm, experiment 27) and PP (80–155 μm, experiment 30) appeared unsuitable for separation. PTFE (10–45 μm, experiment 31) was acceptable, but DNA recovery was low. Moreover, because of its low porosity, introducing LE was difficult and required a vacuum. The most promising material turned out to be UHMWPE (experiments 29 and 32), either with factory hydrophilic modification or after O_2_ plasma modification. Since the manufacturer does not disclose the nature of the hydrophilic modification, there is a possibility of releasing unknown substances into the sample during separation.

## Conclusions

Twenty-eight solid materials and seven structural designs were evaluated for the LE/TE boundary stabilization in ETP. Two device formats were tested: a large device (95 mm diameter) to assess stabilization across wide interfaces, and the miniETP (25 mm diameter) to assess the use of thin barriers. Applicability of the media was judged by successful dye separation combined with high DNA recovery.

The practically applicable stabilization media for the miniETP device were filtration membranes, PU sponge, and nylon fabric used as a barrier. The DNA recovery with these barriers ranged from 70 to 100%. An interesting alternative to the barrier was a combination of a 3D-printed insert filled with a dilute (0.2%) agarose gel. In case of stabilization media for a large ETP device, only two foamed polyethylene polymers with different hydrophilic surface modifications were suitable for dye and DNA separation with recovery from 76 to 90%. The last vital alternative was a PDMA column array. This medium did not stabilize the boundary as well as foamed polymers or sponges, but it enabled the separation of analytes with extremely high molecular weights. The solid LE/TE stabilization media can be an important alternative to gel stabilization in ETP. They can be used mainly in miniETP configuration, where a smaller amount of the sample is analyzed, and the migration path is short. In that configuration, the gel stabilization would be challenging due to the narrow and relatively tall gel. Such a shape of the gel would tend to shrink and collapse. In such a case, a membrane with very large pores can be the first choice. Selection of the stabilization medium should be matched to the analyte’s characteristics (size, charge, adsorption tendency) and the device scale.

## Data Availability

All the relevant data are available on 10.57680/asep.0638102.
